#  Concepts of visual consciousness and their measurement.

**DOI:** 10.2478/v10053-008-0035-y

**Published:** 2008-07-15

**Authors:** Stefan Wiens

**Affiliations:** 1 Department of Psychology, Stockholm University, Stockholm, Sweden2 Psychology Section, Department of Clinical Neuroscience, Karolinska Institute, Stockholm, Sweden

**Keywords:** consciousness, awareness, phenomenology, objective, subjective, blindsight

## Abstract

Although visual consciousness can be manipulated easily (e.g., by visual masking), it is unresolved whether it can be assessed accurately with behavioral measures such as discrimination ability and self-report. Older theories of visual consciousness postulated a sensory threshold and distinguished between subjective and objective thresholds. In contrast, newer theories distinguish among three aspects: phenomenal, access, and reflexive consciousness. This review shows that discrimination ability and self-report differ in their sensitivity to these aspects. Therefore, both need to be assessed in the study of visual consciousness.

When people look at pictures, they usually report that they are consciously aware of the pictures. However, visual consciousness can be manipulated easily with many different methods (Kim & Blake, 2005). For example, when pictures are shown in quick succession or close in space, visibility of the pictures may be reduced and thus masked (Breitmeyer & Öğmen, 2006; Enns & Di Lollo, 2000). As a result, participants may report that they are not consciously aware of the masked pictures (Esteves & Öhman, 1993). Does this self-report demonstrate that the participants were truly unaware and thus unconscious of the pictures? Alternatively, if the participants can discriminate among the pictures better than chance even though they deny awareness, does this demonstrate that the participants were actually aware and thus conscious of the pictures? These questions have been debated for more than a century (Eriksen, 1960; Hannula, Simons, & Cohen, 2005, 2006; Holender, 1986; Merikle, Smilek, & Eastwood, 2001; Schmidt & Vorberg, 2006; Wiens, 2006a, 2006b; Wiens & Öhman, 2007). This debate concerns which measure is best to index visual consciousness. However, in this discussion about a valid measure, the conceptualization of visual consciousness has often been only implicit (Reingold & Merikle, 1990). Therefore, this paper outlines traditional and modern concepts of visual consciousness and discusses whether measures are available that capture these concepts adequately.

## TRADITIONAL THRESHOLD CONCEPTS OF VISUAL CONSCIOUSNESS

Early psychophysics argued for the existence of a sensory or observer threshold (Macmillan & Creelman, 2005). This hypothetical threshold was presumed to be internal to the participant and not directly measurable with response behavior; nonetheless, it determines whether or not a stimulus is sensed. This assumption has been central to research on subliminal perception (Merikle et al., 2001). Researchers assumed the existence of an internal threshold or limen of perceptual awareness and tried to study the degree to which stimulus processing occurs below this threshold (subliminal = below threshold). The reasoning was that if it can be shown that processing occurs below this threshold, and thus in the absence of perceptual awareness, then this would be strong evidence for subliminal perception, as awareness could not be a necessary condition for stimulus processing (Frith, Perry, & Lumer, 1999). This approach is also called dissociation procedure because findings require a dissociation between awareness and another measure (Holender, 1986; Schmidt & Vorberg, 2006). The dissociation procedure has received wide interest in determining whether there is subliminal processing of emotional input (for a general review, see Wiens, 2006a; for a review of brain imaging studies, see Wiens, 2006c).

Although the notion of a sensory threshold has intuitive appeal, contemporary psychophysics has abandoned this concept. Specifically, signal detection theory (SDT) is a more parsimonious model that accounts for many findings without the concept of an internal sensory threshold (Macmillan & Creelman, 2005). SDT assumes an internal continuum of sensory states, which is generally referred to as “strength of evidence” (Pastore, Crawley, Berens, & Skelly, 2003). SDT assumes further that because of internal noise, there is no point on the internal continuum that separates indisputably between the presence and absence of a signal. Accordingly, because of internal background noise, the presence of a signal may evoke a small internal response, whereas the absence of a signal may actually evoke a strong internal response. Therefore, at any position on the internal continuum, only probability statements are possible about whether or not a signal was present. When deciding about what to respond, participants choose a cut-off point on this internal continuum. In a detection task, this criterion determines whether participants respond yes or no. On each trial, participants respond yes if the internal response exceeds this criterion; otherwise, they respond no. If the proportion of yes-responses on signal trials (hits) exceeds the proportion of yes-responses on no-signal trials (false alarms), participants can discriminate between signals and no-signals. Discrimination ability is commonly indexed by d’ (pronounced as d prime). Aside from discrimination ability, SDT allows response biases to be measured, which refer to participants’ tendency to favor one response (e.g., yes-responses) and are commonly indexed by beta or C (Macmillan & Creelman, 2005; Wiens, 2006c).

Although the notion of a sensory threshold does not match contemporary psychophysics, Cheesman and Merikle (1984) distinguished two other threshold concepts in subliminal perception. The objective threshold is the “level at which perceptual information is actually discriminated at a chance level” (p. 391), and the subjective threshold is the “level at which subjects claim not to be able to discriminate perceptual information at better than at a chance level” (p. 391). Several concepts in SDT may be considered as possible candidates for the objective and subjective threshold (Macmillan, 1986; Wiens, 2006c). However, because the theoretical basis of the objective and subjective threshold as well as their measurement are unclear (for discussion, see Wiens, 2006c), the remainder of this paper focuses on modern concepts of visual consciousness, but an attempt is made to subsume traditional threshold concepts under modern theories.

## MODERN CONCEPTS OF VISUAL CONSCIOUSNESS

Many theories of visual consciousness have been postulated. Although these theories differ widely in their conceptualization, it may be possible to distinguish three broad aspects of visual consciousness. These have been referred to as phenomenal, access, and reflexive consciousness (Block, 2001). *Phenomenal consciousness* refers to the experience of conscious content, *access consciousness* refers to content that is accessible, and *reflexive consciousness* refers to a state of introspection about the content of consciousness. For example, people in a movie theater may be experiencing the film (phenomenal consciousness), they have content information available about the characters, plot, and audience (access consciousness), and they are self-aware of being in a movie theater and watching a film (reflexive consciousness). A similar distinction between phenomenal consciousness (first-level affect) and reflexive consciousness (second-level awareness) has been proposed for emotional experience (Lambie & Marcel, 2002; Wiens, 2005).

### Phenomenal consciousness

Phenomenal consciousness refers to the experience of content associated with visual perception (qualia), for example, seeing red as red and green as green. However, the minimal features of phenomenal consciousness have yet to be determined. For example, it is unclear if phenomenal consciousness can exist before features are bound together (e.g., red and table to red table) (Lamme, 2003). A measure of phenomenal consciousness does not need to capture the mechanism of qualia (how it is generated, which is referred to as the hard problem of consciousness). It is sufficient that it assesses whether phenomenal consciousness is present or absent. Self-report is commonly accepted as an accurate index of the presence of phenomenal consciousness. If participants report (verbally or via button press) that they are aware of a picture (e.g., this is a red table), they are considered phenomenally conscious. This notion of self-reported consciousness may correspond to the concept of the subjective threshold (discussed above). But, although reported stimuli may indicate the presence of phenomenal consciousness, it is unclear if unreported stimuli necessarily indicate the absence of phenomenal consciousness.

#### Unreported stimuli show absence of phenomenal consciousness?

Many studies have contrasted conditions in which participants reported the presence versus absence of phenomenal consciousness (Frith et al., 1999). However, self-reported absence of phenomenal consciousness may not actually indicate that phenomenal consciousness is absent. For example, participants may participate in a detection task in which they respond whether or not they are aware of faces (yes or no). According to SDT, the participants place the criterion for yes-responses somewhere on the internal continuum. Although it is conceivable that this criterion placement may capture the threshold for phenomenal consciousness, its placement is arbitrary and is often affected by manipulations of the pay-off matrix (e.g., by rewarding correct responses). In the present example, the participants may vary in their criterion (response bias) of what they consider to be sufficient evidence to report that they see a face. That is, some participants may report that they see a face only if they can clearly see all facial features (conservative response bias), whereas some participants may already report that they see a face if they see two eyes (liberal response bias). Hence, the participants may not differ in their actual phenomenal consciousness, but their self-report differs because of differences in response biases.

To reduce individual differences in criterion placement (response bias), the participants may be instructed on where they ought to place their criterion (e.g., which facial features need to be experienced to report awareness). Alternatively, the participants may be allowed to rate the degree of phenomenal consciousness on a continuous scale. This approach provides evidence about whether self-reported phenomenal consciousness is continuous or binary. For example, studies of visual masking suggest that participants generally rate their phenomenal consciousness on a continuum (Esteves & Öhman, 1993), whereas research on the attentional blink suggests that participants report their phenomenal consciousness to be binary (Sergent & Dehaene, 2004). Nonetheless, although continuous scales of self-reported phenomenal consciousness may be preferable to the arbitrary dichotomization into yes- and no-responses, it is unresolved whether participants who report the lowest level on a continuous scale have no phenomenal consciousness.

A common argument against the notion that self-reported absence of phenomenal consciousness reflects its actual absence has been finding that people can often discriminate visual input even if they deny phenomenal consciousness (Holender, 1986). Indeed, SDT accommodates the observation that unreported stimuli can be discriminated (Haase, Theios, & Jenison, 1999; Macmillan, 1986). The reason is that unreported signals show only that these stimuli fall below the response criterion (misses in SDT terms) and do not necessarily demonstrate that participants cannot differentiate between signals and no-signals (i.e., unreported signals do not demonstrate that d’ = 0). Even if a participant has excellent ability to differentiate signals from no-signals (d’ > 0), many signal trials will be reported as missed if the participant has a conservative response bias (i.e., is unwilling to report awareness). Hence, because unreported signals (misses) do not rule out that the participant could differentiate between signals and no-signals, unreported signals do not appear to be convincing evidence for the absence of phenomenal consciousness.

#### Zero discrimination ability shows absence of phenomenal consciousness?

Many researchers have advocated a definition of the absence of phenomenal consciousness in terms of participants’ inability to discriminate signals from no-signals (i.e., d’ = 0). However, because it is debated how to index discrimination ability, it is possible to argue that any measure (e.g., electrodermal activity) reflects phenomenal consciousness. Unfortunately, this argument would make it logically impossible to study processes in the absence of phenomenal consciousness because any evidence of discrimination ability would, by definition, demonstrate phenomenal consciousness per se (Bowers, 1984). However, even if only standard behavioral tasks are allowed, it may be unclear which task is appropriate to assess discrimination ability (Duncan, 1985). For example, ability to discriminate among masked pictures might be measured with a task in which participants have to choose between two verbal labels (e.g., spider or snake). However, it is possible that although the participants may not be able to label the targets, they might be able to discriminate correctly between the two categories if no explicit labels are given (e.g., darker vs. lighter). If so, a discrimination task with explicit labels might be considered an insensitive and thus invalid measure of awareness (Lovibond & Shanks, 2002). In fact, this question about appropriate measurement (i.e., operationalization) is already evident in the original studies by Cheesman and Merikle (1986) , in which performance on either a simple yes-no word detection or a more complex word-identification task was used interchangeably as an index of awareness.

Further, absence of phenomenal consciousness is often defined as chance performance (for review, see Wiens, 2006a). However, this is an attempt to prove the null and requires strong statistical power to avoid a Type 2 error (falsely concluding that non-significant performance reflects absence of phenomenal consciousness). Because studies vary in the number of trials and significance criterion, non-significant findings have been challenged on lack of statistical power (Hannula et al., 2005). Further, to obtain a reliable index of discrimination ability, many trials are required at low visibility. As a consequence, poor performance may reflect lack of motivation rather than absence of discrimination ability (Merikle & Daneman, 2000).

In general, these arguments illustrate the challenge to find a measure that is exhaustive and thus captures all aspects of phenomenal consciousness completely (Merikle & Reingold, 1998). Accordingly, if a measure is not exhaustive, any purportedly unconscious effects might actually be due to conscious processes that were missed by the (inexhaustive) measure of awareness (Wiens & Öhman, 2007). To avoid this discussion, two alternative approaches have been suggested. First, Reingold and Merikle (1988) proposed to compare the relative sensitivity of two measures. If it is assumed that one measure (direct measure) is at least as sensitive to conscious processes as a second measure (indirect measure), then greater effects on the indirect than direct measure would demonstrate that these effects were due to unconscious processes. Because this approach compares only relative sensitivities between two measures, it is not necessary that either measure is an exhaustive measure of awareness (Schmidt & Vorberg, 2006). Second, it may be beneficial to treat awareness as a continuous variable and to study dose-response relationships between changes in awareness and other variables (Wiens, 2006a). If research shows that changes in awareness differ qualitatively from changes in other variables (Merikle & Cheesman, 1987), these findings would suggest that changes in other variables occur independently from changes in awareness (Schmidt & Vorberg, 2006). These two approaches are worth pursuing because they attempt to identify processes that are independent from changes in awareness (Schmidt, this volume). However, they are not helpful for the present discussion because they say little about the status of phenomenal consciousness per se based on discrimination ability and self-report. 

In fact, the main conceptual issue in indexing phenomenal consciousness with discrimination ability is that this ignores the principally subjective nature of phenomenal consciousness. That is, it seems more relevant to measure what people experience subjectively rather than what they can discriminate objectively (Bowers, 1984; Wiens & Öhman, 2002). By analogy, the experience of pain per se is not captured by people’s ability to discriminate pain stimuli objectively, but only by the fact that they experience them subjectively as painful (Wiens, 2006b).

#### Phenomenal consciousness despite zero discrimination ability?

 Most researchers would probably agree that phenomenal consciousness is absent if there is strong evidence (e.g., relevant discrimination task, sufficient power) that discrimination ability is absent (d’ = 0). However, several researchers suggest that self-report and discrimination ability may not be sensitive enough to index phenomenal consciousness (Block, 2001, 2005b; Lamme, 2003, 2006; for the notion of micro-consciousness, see Zeki, 2003). Accordingly, d’ = 0 may not be sufficient to guarantee absence of phenomenal consciousness. Block (2001) illustrates this point with a task modeled after the Sperling (1960) study on iconic memory. When arrays of letters (e.g., 3 x 3) are presented briefly, participants report that they can see all the letters, although they can actually report and discriminate only a few of them. According to Block, this example demonstrates that phenomenal consciousness is much broader than what self-report and discrimination ability suggest. Similarly, Lamme (2003) proposed that processes are either phenomenally unconscious or conscious. If the processes that are phenomenally conscious are attended, they result in self-report and discrimination ability. (Lamme talks about “conscious report,” but in his examples on change blindness, he uses discrimination performance as evidence for phenomenal consciousness.) Thus, many processes are phenomenally conscious but do not affect self-report and discrimination ability unless they are attended.

Although interesting, this model has two drawbacks. First, attention is considered secondary to phenomenal consciousness; however, because in lower animals there is stronger evidence for attention than phenomenal consciousness, these data suggest that attention is more basic than consciousness. Notably, Dehaene and his colleagues proposed a model that considers consciousness to be secondary to attention (Dehaene, Changeux, Naccache, Sackur, & Sergent, 2006). Accordingly, some processes are always unconscious even if attended (*subliminal* processes). Because attention may facilitate these processes even if they remain unconscious, these findings demonstrate that attention is independent from consciousness (Naccache, 2005). Further, processes that have the potential to become conscious are considered conscious (phenomenally) if they are in the current focus of attention and *preconscious* if they are currently outside the focus of attention. In sum, attention either selects the aspect of phenomenal consciousness that is available to self-report and discrimination ability (Lamme), or mediates phenomenal consciousness (Dehaene).

Second, because this view implies that any behavioral measure may be too insensitive to index the absence of phenomenal consciousness, it seems difficult to validate this model because even complete absence of self-report and discrimination ability (d’ = 0) may not necessarily demonstrate absence of phenomenal consciousness. However, it has been proposed that recurrent processing may be necessary and sufficient for phenomenal consciousness (Block, 2005b; Lamme, 2006). Recurrent processing refers to feedback loops between brain areas and is distinguished from the feedforward sweep, which is the direct activation of cells in successive stages of the cortical hierarchy (Lamme, 2004). In fact, Lamme (2006) proposed that behavioral measures ought to be abandoned as the gold standard in favor of neural measures (i.e., recurrent processing). Lamme argues that phenomena of split brain, neglect/extinction, change blindness, inattentional blindness, and attentional blink may be failures of processes other than consciousness (e.g., failures of language, attention, and memory). If recurrent processing is present in these conditions, it would support the notion that these patients have phenomenal consciousness. Further, if behavioral effects of visual input can be shown to correspond more closely to the percept (phenomenal consciousness) rather than its physical properties, these findings would support the presence of phenomenal consciousness. Breitmeyer, Ro, and Singhal (2004) studied this latter question for masked color priming. Participants were shown blue, green, and white disks that were masked with blue and green rings. Due to this meta-contrast masking, the participants could not discriminate among the blue, green, and white disks. Nonetheless, when the participants had to name the color of the rings as quickly as possible (blue or green), they were faster to name the color of the green than the blue rings when these were preceded (primed) by a white disk. In contrast, when the participants labeled the color of the disk (blue, green, or white), they tended to mislabel the white disks more often as blue than green. Because white was physically closer to green than blue, these findings suggest that unconscious color priming in the absence of discrimination ability follows the physical properties of the prime rather than the percept. According to Lamme’s model, these findings suggest that masked color priming is truly phenomenally unconscious. In support, research suggests that visual masking eliminates recurrent processing (Lamme, 2004), which Lamme considers to be necessary and sufficient for phenomenal consciousness.

#### No phenomenal consciousness despite good discrimination ability?

The most difficult situation in deciding about the status of phenomenal consciousness is when discrimination ability is present (d’ > 0) although participants report no phenomenal consciousness. As discussed above, it seems unreasonable to conclude that any evidence of discrimination ability per se is proof of phenomenal consciousness, because lower animals and machines can perform discrimination tasks without apparent phenomenal consciousness. Similarly, because conclusions about d’ > 0 are often based on significance testing, and because sufficient power may result in significance even for a tiny effect size, it also seems unreasonable to conclude that any deviation from nill (e.g., d’ > .01) in itself indicates phenomenal consciousness (Wiens, 2006a).

However, observations of blindsight in humans clearly challenge the idea that d’ > 0 reflects phenomenal consciousness (Pöppel, Held, & Frost, 1973; Weiskrantz, 1986; Weiskrantz, Warrington, Sanders, & Marshall, 1974). These patients have damage to the primary visual cortex and are typically considered to be clinically blind in the damaged visual field. However, experiments have shown that these patients can localize light flashes accurately in their damaged visual field. Critically, patients can localize light flashes accurately but categorize them as blanks (i.e., no light flash) when given the option to do so. Indeed, blindsight patients commonly report that they have no phenomenal consciousness of any light flashes in the damaged visual field and need to be encouraged to guess the location of the light flashes. Therefore, patients perform well in localizing light flashes even though they deny phenomenal consciousness of the lights in their damaged visual field (they exhibit blindsight). Notably, patients vary in their degree of blindsight. That is, few patients report absolutely no phenomenal consciousness whatsoever (Type 1), whereas the majority of patients report some vague experiences (Type 2) (Weiskrantz, 1997).

Extensive research on humans and monkeys suggests that alternative explanations for the observed findings are unlikely (Azzopardi & Cowey, 1997; Cowey, 2004; Cowey & Stoerig, 1995; Stoerig, Zontanou, & Cowey, 2002). This research has provided good evidence that targets in the damaged visual field are not classified as blanks merely because they may appear somewhat less visible than targets in the undamaged visual field (and thus fall below the response criterion). That is, when target contrast in the undamaged visual field was reduced considerably (which also reduced localization performance), targets in the damaged visual field were still classified as blanks (even though localization performance was excellent). Hence, although blindsight patients may exhibit conservative response biases, their detection performance remains affected after controlling for the confounding effect of response biases (Azzopardi & Cowey, 1997). Further, the findings do not appear to be due to a lower frequency or to different outcomes (e.g., no rewards) associated with targets in the damaged than undamaged field (for further discussion, see Wiens, 2006c). Taken together, these findings suggest that blindsight patients (at least of Type 1) do not have phenomenal consciousness.

According to Lamme’s (2003) model, blindsight patients ought to have phenomenal consciousness if they show recurrent processing. However, Lamme (2006) suggests that blindsight is one of the manipulations that eliminate phenomenal consciousness (i.e., no recurrent processing). Aside from blindsight, Lamme believes this to be true for visual agnosia, backward masking, dichoptic masking, transcranial magnetic stimulation, and binocular rivalry. Lamme postulates that these conditions show no phenomenal consciousness (i.e., no recurrent processing), whereas conditions of split-brain, neglect/extinction, change blindness, inattentional blindness, and attentional blink show phenomenal consciousness (recurrent processing) that cannot be reported. The main argument for this distinction is that in the first case (e.g., blindsight), information remains completely inaccessible, whereas in the second case (e.g., inattentional blindness), information can potentially be accessed but is currently not because of manipulations in attention, memory, and language. For example, because change blindness and inattentional blindness disappear when participants attend to the relevant location of the changes, these conditions demonstrate phenomenal consciousness. In contrast, blindsight has no phenomenal consciousness because no manipulation seems to restore self-report of phenomenal consciousness, and because visual input in the lesioned field does not elicit behavior spontaneously (only in forced-choice tasks). 

One difficulty with this argument is that whereas self-reported absence of phenomenal consciousness is considered as too insensitive in cases such as inattentional blindness, it is now allowed as evidence in blindsight. Also, it is unclear why blindsight may not also be due to difficulties in attention; that is, blindsight patients may experience phenomenal consciousness but cannot report it because they cannot attend to it. Last, Lamme refers to observations that visual input does not elicit behavior spontaneously. This argument mirrors the proposal of a close relationship between phenomenal consciousness and the functional effects of consciousness (i.e., access consciousness, discussed below). If so, this argument seems inconsistent with the suggestion that phenomenal consciousness is separate from other aspects of consciousness.

Taken together, in the Lamme model, recurrent processing indicates phenomenal consciousness, and self-report and discrimination ability cannot be used to decide about absence of phenomenal consciousness. Although there is strong evidence that recurrent processing may be necessary for phenomenal consciousness, it is unclear if it is sufficient. Unfortunately, because there is no external evidence other than recurrent processing itself to demonstrate phenomenal consciousness, it is not apparent how this model can be tested scientifically.

#### Phenomenal consciousness is phenomenal

 Introspection supports the notion of a phenomenal consciousness that is much richer than is captured by self-report and discrimination ability. For example, in discussing the task in the Sperling (1960) study, Block (2001) argues that participants experience phenomenal consciousness of all the letters, but they cannot report and discriminate more than a few (see Lamme, 2003, for a similar argument). However, despite some face validity, common sense does not support the notion that phenomenal consciousness may include experiences of which people are completely unaware themselves (Rosenthal, 2002). Accordingly, Dehaene et al. (2006) propose that the impression of a detailed phenomenal consciousness is an illusion. Because people can potentially attend to details, they falsely believe that they actually have phenomenal consciousness of all of them. To support this perspective, Dehaene et al. refer to cases of inattentional blindness and change blindness. There, large changes in visual input are not detected unless they are attended. 

However, an alternative explanation is that the participants in the Sperling (1960) task may have phenomenal consciousness of the letters, but that this phenomenal consciousness does not include the experience of the identities of the letters (Rosenthal, 2002). Similarly, when shown pictures, participants may rapidly experience that they are viewing a landscape or a city skyline (VanRullen & Thorpe, 2001). That is, within a brief interval people may experience the gist of a picture rather than its details. In higher-order thought (HOT) theory, these experiences are referred to as thoughts (e.g., landscape), and these thoughts define phenomenal consciousness (Rosenthal, 2002). Notably, HOTs are not limited to complex and complicated processes (e.g., I am aware that I am looking at a picture of a landscape), but apply also to simpler processes (e.g., This is a landscape). In sum, HOTs fit intuitive notions of what consciousness means and seem more parsimonious than an information processing mechanism that generates phenomenal consciousness with many details of which only a subset is available to affect discrimination ability and self-report. 

### Access consciousness

 The concept of access consciousness is best understood in terms of the function of consciousness. Because the brain consists of numerous specialized networks that operate in parallel, there needs to be a process that integrates this flow of information. Many theories state that consciousness is the agent that is responsible for this integration of information (Baars, 2002, 2005). Block (2001) assigned the term access consciousness to describe this complex process of information integration within the brain. In support of its importance, access consciousness seems to be required to generate unusual, novel, and spontaneous behavior (Dehaene & Naccache, 2001). There are many theories about the neural mechanisms of access consciousness (Baars, 2002). For example, Dehaene and Naccache (2001) propose that global workspace neurons (mainly in prefrontal and anterior cingulate cortices) mediate access consciousness through attentional amplification of specialized networks (e.g., visual cortex). Thus, access consciousness is not localized to a particular brain region but is reflected in dynamic patterns of brain activation. 

Because access consciousness refers to a complex process of information integration, discrimination ability seems to be the measure of choice, whereas self-report may be less sensitive. Thus, in a task that is novel or requires strategic behavior, evidence of discrimination ability may be taken as good evidence for access consciousness even in the absence of self-reported unawareness. However, task complexity may not be a relevant criterion to infer access consciousness. For example, reading is a complex task that may become completely automatic and require little or no access consciousness to result in semantic priming (Dehaene et al., 1998).

Because the concept of the objective threshold (discussed above) focuses on discrimination ability, it may be closest to access consciousness. However, access consciousness cannot be inferred from discrimination ability per se but depends on the context that requires novel or strategic behavior. To illustrate, although blindsight allows for accurate discrimination ability (i.e., localization of targets in the damaged visual field), this is not considered as sufficient evidence for access consciousness, as food-deprived monkeys with blindsight do not reach for food presented in their blind field (Cowey, 2004). Thus, information in the blind field is not directly accessible even though it can affect discrimination ability.

Although access consciousness may be conceptualized as separate from phenomenal consciousness (Block, 2001), this point is currently debated. For example, Baars and Laureys (2005) argue that phenomenal and access consciousness reflect the same process: Information that is accessed is phenomenally conscious. As mentioned above, Dehaene et al. (2006) share this view but distinguish further among subliminal, preconscious, and conscious processes. Processes are subliminal if they can never be accessed, preconscious if they can potentially be accessed but are currently not (because they are not attended), and conscious if they are currently accessed (and thus accompanied by phenomenal consciousness). Dehaene et al. suggest that preconscious processes are not accompanied by phenomenal consciousness (because they are not accessed). In contrast, as discussed in the section on phenomenal consciousness, this view is not shared by Block (2005b) and Lamme (2003) . As paraphrased by Block (2005a) , “the content of experience can exist in the back of the head without access to it in the front of the head” (p. 270). 

### Reflexive consciousness

Reflexive consciousness refers to the self-awareness of phenomenal consciousness (e.g., I am aware that I am reading an article on consciousness). Reflexive consciousness has also been referred to as introspective or monitoring consciousness (Rosenthal, 2002) to reduce potential misunderstanding, as the term reflexive implies reflex-like processing even though its actual meaning is closer to reflective processing. A convincing illustration of the independence of reflexive consciousness from phenomenal and access consciousness may be observations of mind wandering. People may experience mind wandering without noticing that their minds are wandering (Schooler, 2002). That is, people may have phenomenal consciousness of ongoing events and respond to these events (access consciousness) without any self-awareness (no reflexive consciousness) of what they are doing or looking at. Thus, reflexive consciousness may be closest to the process of noticing. Because it has been argued that the subjective threshold (discussed above) ought to capture what people notice (Bowers, 1984), it may be subsumed under reflexive consciousness. Indeed, it is possible to argue that processes are unconscious unless they result in reflexive consciousness (Dienes, 2004).

Because reflexive consciousness is introspective, self-report is the measure of choice. Unfortunately, many studies have interviewed participants only at the end of the experiment about their consciousness during the experiment. This post-hoc assessment may confound reflexive consciousness with memory. So, unless reflexive consciousness is defined as awareness that can be recalled some interval (e.g., minutes) after the actual event, the measure should be assessed immediately after the event (Lovibond & Shanks, 2002). Although self-report has intuitive appeal, it has been criticized because participants may differ in their introspective report even though their discrimination ability may be similar. However, because reflective consciousness is, by definition, introspective and thus relies on self-report, it is irrelevant whether or not participants can actually discriminate visual input.

Unfortunately, phenomenal consciousness is sometimes described to include features of reflexive consciousness. This makes it difficult to separate the three aspects of visual consciousness conceptually. This difficulty can be illustrated in the discussion of blindsight. Patients with blindsight report that they are not aware of any targets, but when forced to do so, they can discriminate the location of these targets. Thus, their reflexive consciousness suggests absence of phenomenal consciousness. In this research, patients are allowed to report the apparent absence of targets by classifying targets as blanks (empty trials). This option is commonly referred to as a “commentary key,” which implies reflexive consciousness. However, because blindsight has also been demonstrated in monkeys, blindsight may not indicate impaired reflexive consciousness (i.e., monkeys may not have to be self-aware that they experience blanks) (Block, 2005b). Instead, patients may have no phenomenal consciousness of the targets in the damaged visual field and just report it as such (e.g., there is nothing there). Further, they may have no ability to access and use the information to control behavior (no access consciousness). This example illustrates that reflexive consciousness is best reserved for processes in which participants monitor their own awareness introspectively.

### Conclusion

There is no single behavioral measure that captures all aspects of visual consciousness, but different measures can be used to assess different aspects of visual consciousness. Discrimination ability may be useful in indexing access consciousness, for example in strategic and novel behavior. Self-report is an excellent tool to assess the presence of reflexive and phenomenal consciousness. However, evidence for the presence of any aspect of consciousness may be easier to obtain than for its absence. Thus, it is a matter of debate first, whether the absence of discrimination ability and self-report demonstrates absence of phenomenal consciousness, and second, whether the presence of discrimination ability despite self-reported unawareness indicates phenomenal consciousness. As illustrated by research on blindsight, patients show good discrimination ability on localization tasks, but report blanks on detection tasks. Thus, patients indicate absence of phenomenal consciousness despite findings of their preserved ability to localize the targets. To distinguish behaviorally between the apparent absence and presence of different aspects of visual consciousness, I advocate the concurrent use of behavioral measures of discrimination ability and self-report. Here, self-report refers to measures that are methodologically sound (e.g., online visibility ratings rather than post-experimental interviews). When combined with brain imaging methods, these measures will contribute to our understanding of the conditions that are necessary and sufficient for various aspects of visual consciousness and will help determine whether various aspects of visual consciousness have independent neural mechanisms.
